# Efficacy and safety of Qiangli Dingxuan tablet combined with amlodipine besylate for essential hypertension: a randomized, double-blind, placebo-controlled, parallel-group, multicenter trial

**DOI:** 10.3389/fphar.2023.1225529

**Published:** 2023-07-10

**Authors:** Jianguo Lin, Qingqing Wang, Dongsheng Zhong, Jinju Zhang, Tianhui Yuan, Hui Wu, Bin Li, Shuangdi Li, Xiaoliu Xie, Dongqing An, Yue Deng, Shaoxiang Xian, Xingjiang Xiong, Kuiwu Yao

**Affiliations:** ^1^ Guang’anmen Hospital, China Academy of Chinese Medical Sciences, Beijing, China; ^2^ Tianjin University of Traditional Chinese Medicine, Tianjin, China; ^3^ First Affiliated Hospital of Guangzhou University of Chinese Medicine, Guangzhou, China; ^4^ First Affiliated Hospital of Henan University of Chinese Medicine, Zhengzhou, China; ^5^ Affiliated Hospital of Changchun University of Chinese Medicine, Changchun, China; ^6^ Traditional Chinese Medicine Hospital of Xinjiang Uygur Autonomous Region, Urumqi, China; ^7^ Eye Hospital China Academy of Chinese Medical Sciences, Beijing, China

**Keywords:** traditional Chinese medicine, Qiangli Dingxuan tablet, hypertension, randomized controlled trial, classic herbal formula, integrated traditional Chinese and western medicine

## Abstract

**Background:** Hypertension, a major cardiovascular risk factor, severely impacts patients’ quality of life. Qiangli Dingxuan tablet (QDT) is a formally approved Chinese patent medicine, which has been widely used as an adjunctive treatment for hypertension. This study aimed to investigate the antihypertensive efficacy and safety of QDT combined with amlodipine besylate in patients with essential hypertension.

**Methods:** In this randomized, double-blind, placebo-controlled, parallel-group, multicenter trial conducted in China, patients diagnosed with grade 1 to 2 essential hypertension were randomly assigned in a 1:1 to the treatment of QDT or placebo for 12 weeks, alongside their ongoing treatment with amlodipine besylate. The primary outcome was the change in office blood pressure (BP) from baseline to 12 weeks. In addition, safety analysis included the assessment of vital signs and laboratory values.

**Results:** At baseline, 269 patients were randomly assigned to the QDT group (*n* = 133) or the placebo group (*n* = 136), and there were no significant differences in baseline characteristics between the two groups. The primary outcome based on the full analysis set from baseline to 12 weeks showed that the mean difference in the change of office systolic BP reduction between the two groups was 6.86 mmHg (95%CI, 4.84 to 8.88, *p* < 0.0001), for office diastolic BP, the mean difference in the change of office diastolic BP reduction between the two groups was 4.64 mmHg (95%CI, 3.10 to 6.18, *p* < 0.0001). In addition, traditional Chinese medicine symptom scores were significantly decreased in the QDT group compared with the placebo group. No severe adverse events attributable to QDT were reported.

**Conclusion:** The combination of QDT and amlodipine besylate demonstrates superior efficacy compared to amlodipine besylate monotherapy in the management of essential hypertension. QDT shows potential as an adjunctive treatment for essential hypertension. However, further rigorous clinical trials are warranted to validate these findings.

**Clinical Trial Registration:** [https://clinicaltrials.gov/study/NCT05521282?cond=NCT05521282&rank=1]; Identifier: [NCT05521282]

## 1 Introduction

Hypertension, defined as systolic blood pressure (SBP) ≥140 mmHg or diastolic blood pressure (DBP)≥90 mmHg, is a prevalent chronic condition and a leading modifiable risk factor for cardiovascular disease (CVD) and mortality worldwide. The 2012–2015 China Hypertension Survey revealed that nearly a quarter of Chinese adults (approximately 244.5 million) had hypertension, and over two-fifths (around 435.3 million) had prehypertension. Alarmingly, less than a third of individuals with prehypertension are receiving treatment, and fewer than one in twelve have achieved adequate blood pressure (BP) control (Lu et al., 2017; Wang et al., 2018). Furthermore, every 10 mmHg increase in SBP raises the risk of ischemic heart disease by approximately 30%, About half of all vascular deaths in China are attributable to elevated BP (SBP >120 mmHg), resulting in nearly one million deaths annually in people under 80 years of age (Lacey et al., 2018). Despite many beneficial changes in people’s lifestyles, the incidence and prevalence of hypertension continue to rise in China due to population growth and aging, as well as adverse lifestyles such as unhealthy diets and insufficient physical activity (Zhao et al., 2019). Therefore, achieving tight BP control in hypertensive patients is imperative to prevent cardiovascular morbidity and mortality.

Pharmacological management of hypertension has advanced over the years, yet several limitations remain. One of the primary issues is poor adherence, encompassing failure to initiate medication, adhere to treatment long-term, and take medication as prescribed, which has been identified as a key contributor to suboptimal BP control among hypertensive individuals (Burnier and Egan, 2019; Jiang et al., 2020). Furthermore, antihypertensive drugs may lead to electrolyte imbalances, orthostatic hypotension, angioedema, cough, and other unfavorable reactions, which can compromise patient adherence and hinder treatment success (Shira et al., 2015; Hripcsak et al., 2020; Albasri et al., 2021).

Traditional Chinese medicine (TCM) has been used for over two millennia in China to treat a wide range of illnesses and has yielded notable outcomes. Substantial evidence supports the superior efficacy and safety of combining TCM classic formulas with conventional treatment compared to conventional treatment alone in the management of hypertension (Xiong et al., 2015; Zhang et al., 2020; Lai et al., 2022). The formulation of TCM, Qiangli Dingxuan tablet (QDT) is derived from the ancient formula “Xiong Ma Yin” in “Su Shen Liang Fang."QDT is composed of 5 herbs, including the tuber of *Gastrodia elata* Blume (Tianma), the bark of *Eucommia ulmoides* Oliv. (Duzhong), leaf of *Eucommia ulmoides* Oliv. (Duzhongye), rhizome of *Conioselinum anthriscoides ‘Chuanxiong’* (Chuanxiong), and *Chrysanthemum indicum* L. (Yejuhua). Tianma is the monarch herb that can relieve wind and spasm, suppress liver yang. Yejuhua acts as a minister herb that clears heat and remove toxins, calms the liver Yang. Chuanxiong is a minister herb that invigorates blood circulation, disperses wind, and relieves pain. Duzhong and Duzhongye are the assistant herbs that nourish the liver and kidney, and strengthen the tendons and bones. The combination of these herbs has the potential to soothe the liver, regulate qi, relieve pain, and promote blood circulation. Experiments have shown that these herbs have the effects of regulating the circulatory system, regulating metabolism, regulating intestinal flora, anti-hypertension, anti-ischemia, anti-inflammation, and anti-oxidation (Matias et al., 2016; Bai et al., 2018; Chen et al., 2018; Hu et al., 2020; Sun et al., 2022). Clinical studies have shown that compared with conventional treatment, QDT combined with conventional treatment has a better antihypertensive effect and can reduce the symptoms of vertigo. Currently, QDT is a formally approved Chinese patent medicine by China Food and Drug Administration (approval number: Z61020139), which has been widely used as a complementary treatment for hypertension, hyperlipidemia, and vertigo in China (Zhao, 2018; Li et al., 2019a; Zhang et al., 2021). However, the low quality of existing studies restricts their ability to offer high-quality clinical evidence-based findings (Li et al., 2019b; Xu et al., 2021; Yin and Yan, 2021; Xu, 2022). Therefore, to validate the efficacy and safety of QDT for hypertension treatment, we conducted a randomized, double-blind, placebo-controlled, parallel-group, multicenter trial.

## 2 Methods

### 2.1 Study design

**FIGURE 1:**
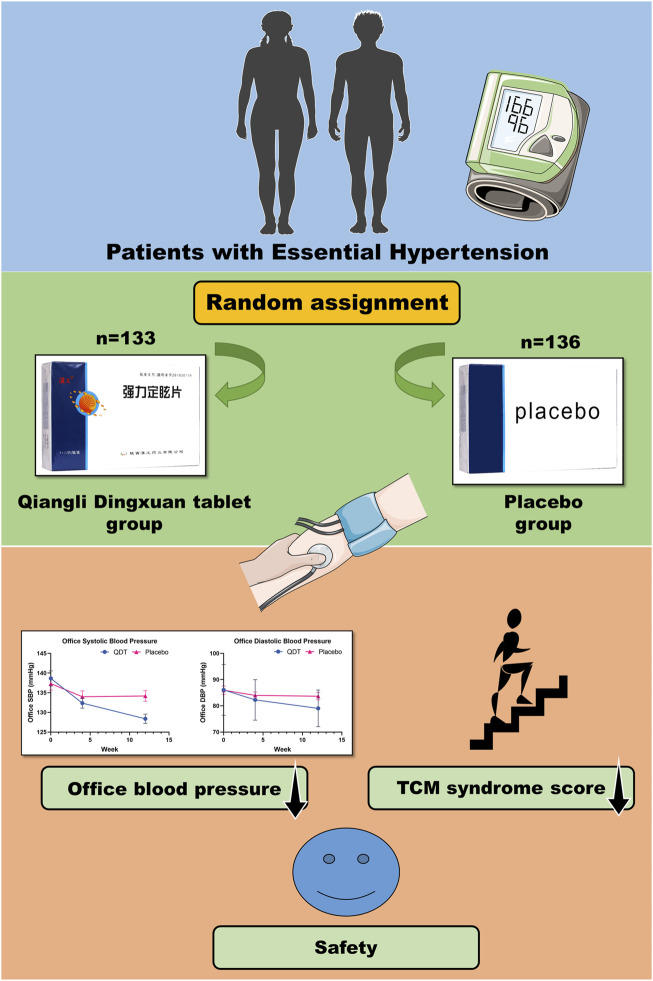
Graphical abstract.

This was a randomized, double-blind, placebo-controlled, parallel-group, multicenter trial, which was done at 5 centers throughout China between March 2021 and June 2022. The trial protocol was approved by the Ethics Committee of Guang ‘anmen Hospital, China Academy of Chinese Medical Sciences (approval number: 2021-008-KY). The trial complied with the Declaration of Helsinki and was registered in ClinicalTrials.gov (Unique identifier: NCT05521282). The reporting in this article follows the Consolidated Standards of Reporting Trials. All participants were required to sign an informed consent form. After confirming that the participants provided informed consent and met the inclusion and exclusion criteria, a random number was assigned to the participants using a randomized system. This study had a double-blind design, and the participants, investigators, and anyone involved in the analysis or interested in the trial were unaware of the trial drug class. Drugs can only be distinguished by drug number (random code) (Figure 1).

### 2.2 Participants

The inclusion criteria: Men and women aged 18–75 years, diagnosed with grade 1 to 2 essential hypertension (SBP, 140–179 mmHg and DBP, 90–109 mmHg) and with TCM syndrome diagnosis of hyperactivity of liver yang (The main TCM symptoms of the patients include vertigo, headache, and impetuosity, and the secondary symptoms include the red face, red eyes, dry mouth, bitterness in the mouth, constipation, etc). Hypertension was diagnosed according to the 2018 Chinese guidelines for the management of hypertension (Writing Group of 2018 Chinese Guidelines for the Management of Hypertension, 2019). TCM syndrome diagnosis standard according to the instructional principle of the latest Chinese herbal medicine to clinical research (Zheng, 2002).

The Exclusion criteria: 1)Patients with secondary hypertension; 2)Patients with severe cardiovascular conditions such as coronary atherosclerotic heart disease, acute exacerbation of chronic heart failure, malignant arrhythmia, valvular heart disease, cardiomyopathy, and other significant cardiovascular disorders; 3)Patients with acute cerebrovascular diseases including cerebral infarction and cerebral hemorrhage; 4)Patients with severe psychological disorders, intellectual disabilities, or language impairments that hinder full cooperation with the study or completion of the study; 5)Patients with known allergies to QDT, excipients, or similar ingredients of the trial drug; 6)Patients with suspected or documented history of alcohol or drug abuse; 7)Pregnant or lactating women, or those who have recently planned or are unwilling to use contraceptive measures.

### 2.3 Interventions

In this study, eligible patients were randomized into either the QDT or placebo group in a 1:1 ratio. Patients in the QDT group were treated with six QDT tablets (0.35 g/tablet; Shaanxi Hanwang Pharmaceutical Co., Ltd., Shaanxi, China), 3 times a day for 12 weeks based on basic treatment. Patients in the placebo group were treated with six placebo tablets (0.35 g/tablet; Shaanxi Hanwang Pharmaceutical Co., Ltd., Shaanxi, China), 3 times a day for 12 weeks based on basic treatment. The basic treatment regimen included taking one tablet of amlodipine besylate once a day (5 mg/tablet; Sinopharm Group Rongsheng Pharmaceutical Co., Ltd., Henan, China), adhering to a low-salt, low-fat diet, abstaining from smoking and alcohol consumption, consuming more vegetables, engaging in moderate exercise, and maintaining a healthy weight. The placebo tablets, composed mainly of starch, mimicked QDT but lacked active ingredients. All study drugs were packaged and labeled according to Chinese laws and regulations and Good Manufacturing Practice (GMP). During the study period, except for the basic treatment drug and the study drug (QDT, placebo), other drugs that have antihypertensive effects and may affect the QDT were prohibited. The investigator meticulously recorded the number of drugs dispensed, utilized, and returned by the patients and evaluated their medication adherence, which was promptly recorded in the case report form.

### 2.4 Preparation for QDT

The QDT (manufacturer: Shaanxi Hanwang Pharmaceutical Co., Ltd., Shaanxi, China; China Food and Drug Administration approval number: Z61020139) is composed of the tuber of *Gastrodia elata* Blume (Tianma), the bark of *Eucommia ulmoides* Oliv. (Duzhong), leaf of *Eucommia ulmoides* Oliv. (Duzhongye), rhizome of *Conioselinum anthriscoides* ‘*Chuanxiong*’ (Chuanxiong), and *Chrysanthemum indicum* L. (Yejuhua). The quality of QDT conforms to the regulations of Chinese Pharmacopoeia (Chinese Pharmacopoeia Commission, 2015). The specific preparation method of QDT can be found in the Chinese Pharmacopoeia. As determined by high-performance liquid chromatography (HPLC), each tablet of this product contains gastrodin (C_13_H_18_O_7_), not less than 0.60 mg. The HPLC fingerprints show that the active ingredients of QDT include gastrodin, gallic acid, 5-hydroxymethyl furfural,p-hydroxybenzyl alcohol, neochlorogenic acid, chlorogenic acid, vanillic acid, p-hydroxybenzaldehyde, pinoresinol diglucoside, sophoricoside, ligustilide, linarin. (Luo et al., 2022). The HPLC fingerprints of QDT can be seen in the Supplementary Material.

### 2.5 Outcome measures

The primary outcome was the change in office BP from baseline after 12 weeks of treatment. The secondary outcomes included blood lipid, homocysteine (HCY), C-reactive protein (CRP), high sensitivity CRP (hs-CRP), and TCM syndrome score.

Upper arm medical electronic sphygmometers certified by international standard protocols (ESH, BHS, and AAMI) were used for office BP measurement. Omron HEM-7124(OMRON Corporation) was mainly used in this study. The same device should be used for the same participants throughout the study. Participants should sit quietly for at least 5 min before taking their BP. BP was measured twice at 2-min intervals and averaged. If two readings of SBP or DBP differ by more than 5 mmHg, they should be measured again, and the average of the three readings should be recorded.

### 2.6 Safety evaluation

Investigators were responsible for recording all adverse events (AE) that occurred during the study. The duration, severity, and causal relationship of each AE to the study drug should be assessed, and the cause, as well as the actions and results taken to address the AE, should be documented. Clinical laboratory safety assessment included blood routine tests, liver function tests, kidney function tests, and urine routine tests.

### 2.7 Sample size estimation

The sample size was determined based on office SBP. In the previous randomized trial (Wang, 2020), SBP was 126 (13.57) in patients with hypertension after amlodipine besylate treatment. This study predicted a 5.5 mmHg reduction in SBP in the treatment group of QDT combined with amlodipine besylate compared with the placebo group with amlodipine besylate alone. The formula calculated that a sample size of 127 patients per group would ensure a power of 90% at a two-sided α level of 0.05. To account for a dropout rate of 20%, the study sample size was increased to 304.

### 2.8 Statistical analysis

Baseline analysis utilized the full analysis set (FAS), which included all subjects randomized to the study based on the intent-to-treat (ITT) principle. Efficacy analysis was based on the FAS and per protocol set (PPS). Subjects who met the inclusion criteria to enter the study and complete treatment and follow-up, with medication compliance between 80% and 120%, complete data of primary outcome, and no major study protocol violations, constituted the PPS of this study. Safety analysis was performed using the safety set (SS), which included all subjects who received at least one treatment after randomization. Data management and statistical analysis were performed using SAS statistical software version 9.4 (SAS Institute, Cary, NC). Continuous data were compared by the group *t*-test or Wilcoxon rank sum test and categorical data by the chi-square test or Fisher exact test. A *p*-value of less than 0.05 was considered statistically significant for all analyses.

## 3 Results

### 3.1 Baseline characteristics of the patients

**FIGURE 2:**
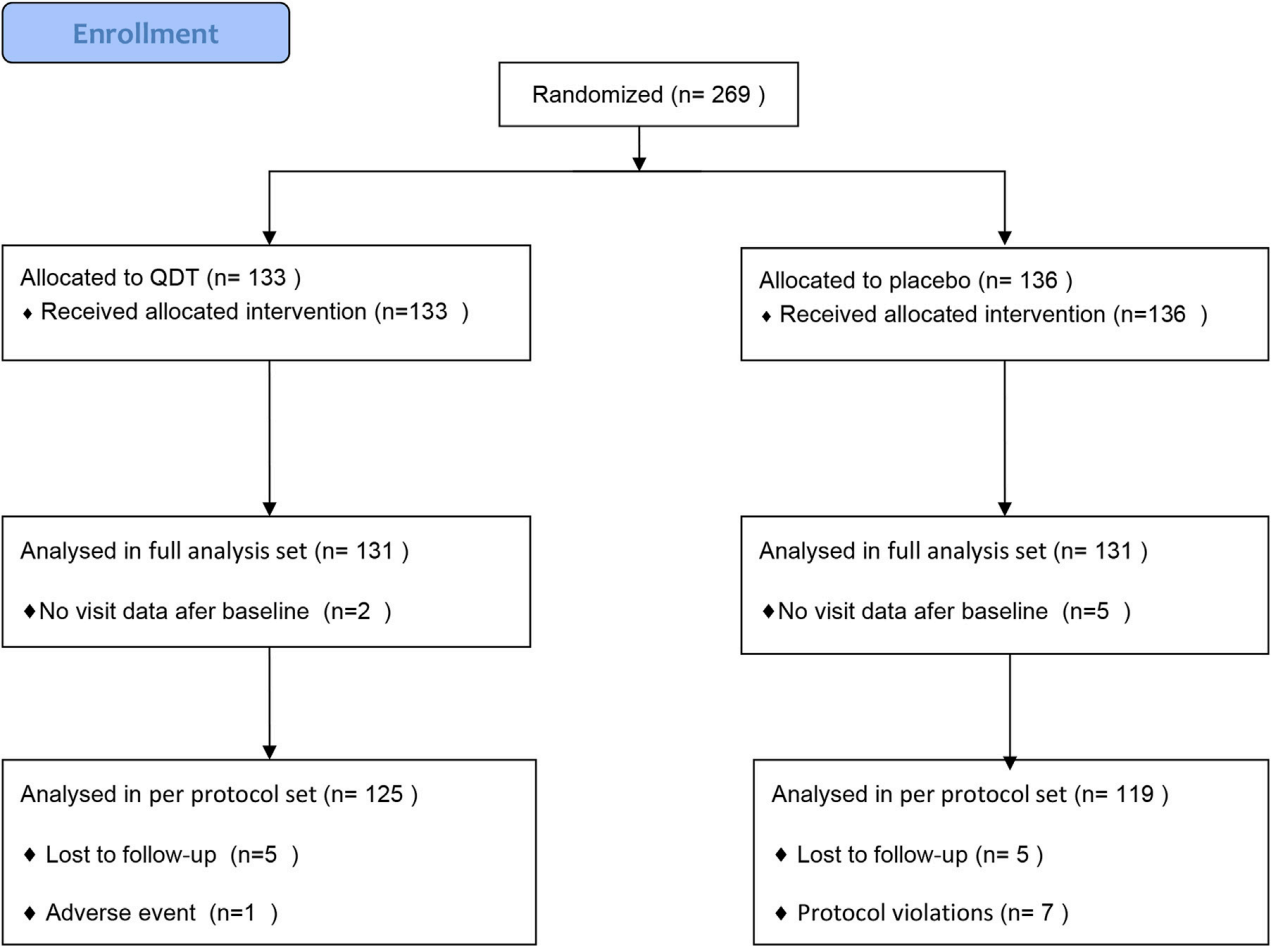
Flow diagram.

269 patients were randomly assigned to the QDT group (*n* = 133) or the placebo group (*n* = 136). 7 participants (2 in the QDT group and 5 in the placebo group) were excluded due to the inability to provide follow-up data, so 262 participants (131 in the QDT group and 131 in the placebo group) were included in the FAS. 18 participants (6 in the QDT group and 12 in the placebo group) were excluded because of loss to follow-up, adverse events, or protocol violations, so 244 participants (125 in the QDT group and 119 in the placebo group) were included in the PPS (Figure 2). The baseline characteristics of the two groups were shown in Table 1. The office SBP/DBP averaged 138.62 (11.09)/86.08 (9.69) mmHg and 137.31 (9.39)/85.96 (9.21) mmHg, respectively. Age, gender, medical history, and cardiovascular risk factors were similar between the two groups (*p* > 0.05).

**TABLE 1:** Baseline Characteristics (analysis based on full analysis set).

Characteristic	QDT group		Placebo group		*p* value
	n (missing)	Mean (SD)/n (%)	n (missing)	Mean (SD)/n (%)	
Men, n (%)	131 (0)	69 (52.67)	131 (0)	65 (49.62)	0.6210
Age, year	131 (0)	54.24 (10.38)	131 (0)	53.77 (11.31)	0.8672
BMI, kg/m^2^	131 (0)	26.04 (3.23)	131 (0)	26.05 (3.52)	0.8175
Hypertension duration, month	131 (0)	61.08 (74.81)	131 (0)	78.35 (80.12)	0.1099
Drinking history, n (%)	131 (0)	44 (33.59)	131 (0)	49 (37.40)	0.5186
Medical treatment history, n (%)	123 (8)	110 (89.43)	121 (10)	104 (85.95)	0.4078
Allergic history, n (%)	131 (0)	3 (2.29)	131 (0)	9 (6.87)	0.0762
Past medical history, n (%)	102 (29)	41 (40.20)	107 (24)	30 (28.04)	0.0636
Office SBP, mmHg	130 (1)	138.62 (11.09)	128 (3)	137.31 (9.39)	0.2780
Office DBP, mmHg	130 (1)	86.08 (9.69)	128 (3)	85.96 (9.21)	0.9164
TC, mmol/L	129 (2)	4.99 (1.17)	127 (4)	5.03 (1.22)	0.9684
TG, mmol/L	128 (3)	1.95 (1.36)	127 (4)	1.99 (1.34)	0.4025
LDL-C, mmol/L	128 (3)	3.11 (0.88)	126 (5)	3.17 (0.84)	0.6132
HDL-C, mmol/L	129 (2)	1.25 (0.33)	125 (6)	1.25 (0.31)	0.3518
Hcy, μmol/L	121 (10)	15.94 (11.58)	120 (11)	13.43 (6.69)	0.2565
CRP, mg/L	113 (18)	1.61 (2.51)	115 (16)	1.81 (2.26)	0.1360

BMI, body mass index; TC, total cholesterol; TG, triglyceride; HDL-C, high-density lipoprotein cholesterol; LDL-C, low-density lipoprotein cholesterol.

### 3.2 Primary outcome

**FIGURE 3:**
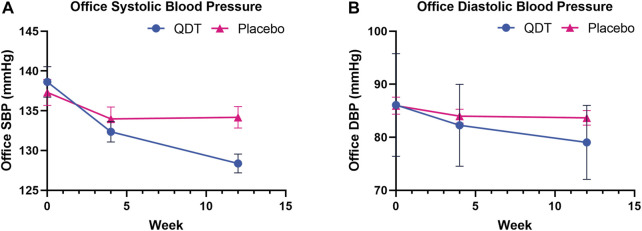
Office systolic/diastolic blood pressure changes at 4 and 12 weeks of treatment. **(A)** Office systolic blood pressure **(B)** Office diastolic blood pressure.

In the FAS, the changes in office SBP from baseline to 12 weeks in the QDT and placebo groups were 9.66 (8.68) mmHg and 2.80 (7.08) mmHg, respectively. The mean difference in the change of office SBP reduction between the two groups was 6.86 mmHg (95%CI, 4.84 to 8.88, *p* < 0.0001). For office DBP, the corresponding changes in the QDT and placebo groups were 6.75 (6.16) mmHg and 2.11 (5.93) mmHg, respectively. The mean difference in the change of office DBP reduction between the two groups was 4.64 mmHg (95%CI, 3.10 to 6.18, *p* < 0.0001). The PPS showed similar trends. The results showed that QDT could effectively reduce office BP in patients with essential hypertension compared with placebo (Table 2; Figure 3).

**TABLE 2:** Primary outcome and secondary outcomes (analysis based on full analysis set).

Outcomes	Visit	QDT group		Placebo group		*p* value
		n (missing)	Mean (SD)	n (missing)	Mean (SD)	
Primary outcome						
Office SBP,mmHg	Baseline	130 (1)	138.62 (11.09)	128 (3)	137.31 (9.39)	0.2780
	4 weeks	131(0)	132.36 (7.47)	131 (0)	133.98 (8.67)	0.0508
	12 weeks	125 (6)	128.38 (6.74)	119 (12)	134.17 (7.49)	<0.0001
	Changes after 4 weeks	130 (1)	6.18 (9.71)	128 (3)	3.41 (7.97)	<0.0001
	Changes after 12 weeks	124 (7)	9.66 (8.68)	116 (15)	2.80 (7.08)	<0.0001
Office DBP, mmHg	Baseline	130 (1)	86.08 (9.69)	128 (3)	85.96 (9.21)	0.9164
	4 weeks	131 (0)	82.25 (7.73)	131 (0)	83.98 (7.57)	0.0690
	12 weeks	125 (6)	79.04 (6.99)	119 (12)	83.66 (7.55)	<0.0001
	Changes after 4 weeks	130 (1)	3.69 (5.92)	128 (3)	2.10 (5.67)	0.0002
	Changes after 12 weeks	124 (7)	6.75 (6.16)	116 (15)	2.11 (5.93)	<0.0001
**Secondary outcomes**						
TC, mmol/L	Baseline	129 (2)	4.99 (1.17)	127 (4)	5.03 (1.22)	0.9684
	12 weeks	90 (41)	4.79 (1.23)	94 (37)	4.66 (1.14)	0.4856
	Changes after 12 weeks	90 (41)	0.31 (0.97)	94 (37)	0.43 (1.16)	0.2062
TG, mmol/L	Baseline	128 (3)	1.95 (1.36)	127 (4)	1.99 (1.34)	0.4025
	12 weeks	90 (41)	1.99 (1.43)	94 (37)	2.31 (2.08)	0.3514
	Changes after 12 weeks	89 (42)	0.07 (0.91)	94 (37)	−0.28 (2.18)	0.2256
LDL-C, mmol/L	Baseline	128 (3)	3.11 (0.88)	126 (5)	3.17 (0.84)	0.6132
	12 weeks	90 (41)	3.07 (0.90)	94 (37)	3.00 (0.85)	0.6094
	Changes after 12 weeks	89 (42)	0.06 (0.77)	93 (38)	0.18 (0.75)	0.1507
HDL-C, mmol/L	Baseline	129 (2)	1.25 (0.33)	125 (6)	1.25 (0.31)	0.3518
	12 weeks	90 (41)	1.28 (0.48)	94 (37)	1.22 (0.37)	0.3242
	Changes after 12 weeks	90 (41)	−0.03 (0.49)	93 (38)	0.05 (0.29)	0.0580
Hcy, μmol/L	Baseline	121 (10)	15.94 (11.58)	120 (11)	13.43 (6.69)	0.2565
	12 weeks	90 (41)	15.36 (11.33)	94 (37)	13.65 (6.46)	0.3703
	Changes after 12 weeks	88 (43)	−0.51 (5.37)	92 (39)	−0.18 (4.81)	0.8379
CRP, mg/L	Baseline	113 (18)	1.61 (2.51)	115 (16)	1.81 (2.26)	0.1360
	12 weeks	88 (43)	1.70 (2.87)	91 (40)	2.55 (6.13)	0.6321
	Changes after 12 weeks	83 (48)	0.24 (2.28)	88 (43)	0.86 (6.28)	0.9070
hs-CRP, mg/L	Baseline	96 (35)	1.16 (2.07)	99 (32)	1.43 (2.09)	0.0151
	12 weeks	84 (47)	1.62 (2.89)	85 (46)	2.40 (6.33)	0.9368
	Changes after 12 weeks	79 (52)	0.26 (2.33)	82 (49)	0.91 (6.49)	0.8798
The total score of TCM syndromes	Baseline	131 (0)	27.21 (5.42)	130 (1)	26.64 (5.37)	0.1561
	12 weeks	125 (6)	22.99 (6.68)	117 (14)	25.44 (7.43)	<0.0001
	Changes after 12 weeks	125 (6)	4.36 (4.29)	116 (15)	1.36 (4.74)	<0.0001

### 3.3 Secondary outcomes

#### 3.3.1 Blood lipid, Hcy, and CRP

Secondary outcomes were shown in Table 2. In the FAS, TC was significantly decreased from baseline to 12 weeks of treatment in the QDT (from 4.99 (1.17) to 4.79 (1.23) mmol/L, *p* = 0.0004) and placebo groups (from 5.03 (1.22) to 4.66 (1.14) mmol/L, *p* < 0.0001). The reductions, however, were not significantly different (*p* = 0.2062) between the two groups. No significant reduction in TG was observed in either group (*p* = 0.2256). LDL-C tended to decrease in the QDT group but was not statistically significant (from 3.11 (0.88) to 3.07 (0.90) mmol/L, *p* = 0.4269), and LDL-C decreased significantly in the placebo group (from 3.17 (0.84) to 3.00 (0.85) mmol/L, *p* = 0.0051). However, the difference between the two groups was not statistically significant (*p* = 0.1507). HDL-C tended to increase in the QDT group but was not statistically significant (from 1.25 (0.33) to 1.28 (0.48) mmol/L, *p* = 0.1243), and HDL-C decreased significantly in the placebo group (from 1.25 (0.31) to 1.22 (0.37) mmol/L, *p* = 0.0002). However, the difference between the two groups was not statistically significant (*p* = 0.0580). In addition, after 12 weeks of treatment, there were no statistically significant differences in the changes in Hcy, CRP, and hs-CRP between the QDT and placebo groups (*p* > 0.05).

#### 3.3.2 TCM symptom score

In the FAS, the changes in the total score of TCM syndromes from baseline to 12 weeks in the QDT and placebo groups were 4.36 (4.29) and 1.36 (4.74), respectively. The mean difference in the total score of TCM syndromes between the two groups was statistically significant (*p* < 0.0001) (Table 2). In addition, among the specific TCM symptoms, QDT was effective in improving dizziness (*p* < 0.0001), impetuosity (*p* < 0.0001), insomnia (*p* = 0.0001), and tinnitus (*p* = 0.0001) (Table 3).

**TABLE 3:** Changes in TCM syndrome scores after 12 weeks of treatment (analysis based on full analysis set).

Outcomes	Mean (SD)		*p* value
	QDT group n (missing) = 125 (6)	Placebo group n (missing) = 116 (15)	
Dizziness	0.33 (0.47)	0.04 (0.43)	<0.0001
Headache	0.34 (0.61)	0.16 (0.52)	0.0099
Impetuosity	0.37 (0.52)	0.04 (0.52)	<0.0001
Aching lumbus	0.18 (0.45)	0.08 (0.33)	0.0521
Limp knees	0.06 (0.38)	0.06 (0.24)	0.9601
Sphoria with feverish sensation in chest, palms and soles	0.20 (0.46)	0.03 (0.34)	0.0013
Head heavy as if swathed	0.22 (0.43)	0.06 (0.40)	0.0024
Chest stuffiness	0.14 (0.34)	0.07 (0.37)	0.0968
Vomiting of phlegm-drool	0.09 (0.28)	0.03 (0.23)	0.1113
Chilly sensation and the cold limbs	0.10 (0.30)	0.04 (0.24)	0.0595
Flushed face	0.11 (0.34)	0.07 (0.34)	0.2836
Red eyes	0.18 (0.40)	0.08 (0.33)	0.0255
Dry mouth	0.30 (0.60)	0.13 (0.57)	0.0081
Bitterness in the mouth	0.24 (0.45)	0.07 (0.39)	0.0027
Constipation	0.15 (0.49)	0.10 (0.50)	0.5433
Hematuria	0.14 (0.37)	0.03 (0.43)	0.0076
Palptation	0.14 (0.37)	0.04 (0.31)	0.0106
Insomnia	0.30 (0.51)	0.05 (0.54)	0.0001
Tinnitus	0.23 (0.44)	0.01 (0.36)	0.0001
Amnesia	0.14 (0.50)	0.06 (0.42)	0.2892
Bland taste in the mouth	0.14 (0.37)	0.01 (0.31)	0.0044
Low food intake	0.05 (0.28)	−0.02 (0.26)	0.0754
Shortness of breath	0.06 (0.29)	0.05 (0.26)	0.9215
Nycturia	0.16 (0.39)	0.07 (0.32)	0.0612

### 3.4 Adverse events

The AE was presented in Table 4. Based on the SAS, the QDT group has 8 patients (8 cases, including 1 case of the gastrointestinal system, 3 cases of the urinary system, 3 cases of the endocrine system, and 1 case of other systems) with AE occurred, and the placebo group has 9 patients (13 cases, including 8 cases of the blood system, 1 case of liver system, 1 case of the gastrointestinal system, 1 case of the urinary system, and 2 cases the endocrine system) AE occurred. Relevance judgment through AE and test drugs, the QDT group has 5 patients (5 cases, including 1 case of the gastrointestinal system, 1 case of the urinary system, 2 cases of the endocrine system, and 1 case of other systems) with adverse reactions. The placebo group has 3 patients (3 cases, including 2 cases of the blood system, and 1 case of the gastrointestinal system) with adverse reactions. There was no significant difference in the incidence of AE and adverse reactions between the two groups (*p* > 0.05). No significant differences were observed in changes in blood routine test, urine routine test, liver function, and renal function (*p* > 0.05) (Supplementary Material).

**TABLE 4:** Adverse events were reported during this study, n (%) (analysis based on safety analysis set).

	QDT group	Placebo group	*p* value
All adverse events	8 (6.02)	9 (6.67)	0.8268
The blood system	0 (0.00)	8 (5.93)	0.0070
The liver system	0 (0.00)	1 (0.74)	1
The gastrointestinal system	1 (0.75)	1 (0.74)	1
The urinary system	3 (2.26)	1 (0.74)	0.3685
The endocrine system	3 (2.26)	2 (1.48)	0.6828
Other systems	1 (0.75)	0 (0.00)	0.4963

## 4 Discussion

To the best of our knowledge, this is the first randomized, double-blind, placebo-controlled, multicenter trial of QDT for the treatment of essential hypertension. After 12 weeks of treatment, office SBP and DBP were significantly lower in the QDT group than in the placebo group. The mean difference in office SBP reduction between the two groups was 6.86 mmHg (95%CI, 4.84–8.88), and the mean difference in office DBP reduction was 4.64 mmHg (95%CI, 3.10–6.18). In addition, QDT can significantly improve TCM symptoms, especially dizziness, impetuosity, insomnia, and tinnitus. The treatment with QDT for 12 weeks did not result in serious AE in patients with essential hypertension. Our study indicates that QDT is a relatively safe medication that can be used as an adjunctive treatment for hypertension under the guidance of a TCM practitioner.

QDT is a formally approved Chinese patent medicine, which has been widely used as an alternative approach for hypertension and vertigo in China. Previous meta-analysis (Ji et al., 2022) showed that QDT combined with conventional treatment was superior to conventional treatment alone in reducing BP and improving clinical efficacy. Similarly, a meta-analysis of 27 studies involving 2,766 patients with vertigo showed that QDT combined with Western medicine could significantly increase cerebral blood flow, reduce BP, improve vertigo and headache symptoms, and had few adverse reactions (Zhang et al., 2022). However, due to the low level of evidence of the included studies, such as low methodological quality (no description of the generation of randomized sequence, blinding, selective outcome reporting, other biases, etc.), small sample size, and incomplete safety reporting. Therefore, although QDT is a formally approved TCM and is commonly used to treat symptoms related to hypertension, its efficacy and safety in reducing BP remain uncertain. As the first placebo-controlled trial of QDT, our study shows that QDT has a significant BP lowering effect in patients with essential hypertension and has good safety.

The goal of hypertension treatment is not only to reduce BP itself, but also to improve the quality of life of patients and reduce the morbidity and mortality of cardiovascular and cerebrovascular diseases. TCM has the advantage of holistic treatment, which can improve symptoms and treat diseases by correcting the imbalance of the human body’s environment (Huang et al., 2021). In this study, we found that QDT could significantly improve the TCM symptoms of hypertensive patients, including dizziness, impetuosity, insomnia, tinnitus, etc., which is particularly important for improving the quality of life of patients. In addition, this study found that QDT had the potential ability to improve blood lipids, although no statistical difference was shown. Therefore, it is hoped that future clinical studies will further verify it.

Hypertension is categorized as “vertigo” and “headache” in TCM, and its main pathogenesis involves the imbalance of Yin and Yang in the liver and kidney (Wang et al., 2014). TCM offers comprehensive and multi-target regulation for hypertension treatment, utilizing its all-round, multi-component, and multi-pathway approach. (Xiong et al., 2013; Li et al., 2021). QDT is composed of 5 herbs, including Tianma, Yejuhua, Duzhong, Duzhongye, and Chuanxiong. We previously found through bioinformatics that the mechanism of QDT in the treatment of hypertension may be related to the PI3K-Akt signaling pathway, Ras signaling pathway, calcium signaling pathway, and cAMP signaling pathway. At the same time, quercetin, kaempferol, acacetin, and syringetin may be the effective components of QDT (Lin et al., 2021). Gastrodin is the main active ingredient obtained from Tianma. Liu et al. (Liu et al., 2015) found that gastrodin (100 mg/kg) injected intraperitoneally for 4 weeks can reduce SBP in spontaneously hypertensive rats (SHR), and the mechanism was related to the inhibition of aldosterone (ALD) and angiotensin II (Ang II) expression by activation of PPARγ. Chen et al. (Chen et al., 2021) demonstrated that gastrodin had a positive effect on the cell viability of homocysteine-induced human umbilical vein endothelial cells through PI3K/Akt/eNOS and Nrf2/ARE pathways, resulting in a significant decrease in malondialdehyde (MDA), lactate dehydrogenase (LDH) and reactive oxygen species (ROS) levels and an increase in nitric oxide (NO) content. In addition, gastrodin can cause vasodilation in the thoracic aorta. Neochlorogenic acid is one of the effective components of QDT. Neochlorogenic acid inhibited migration and proliferation of vascular smooth muscle cells by inhibiting FAK/small GTPase protein, PI3K/Akt and ras related signals, thus playing an anti atherosclerosis role (Yang et al., 2022). Lignans were the effective fraction of Duzhong for antihypertension. Ouyang et al. (Luo et al., 2010; Li et al., 2014) confirmed that the antihypertensive effect of lignans may be related to the regulation of renin-angiotensin system, direct arterial diastole, and regulation of NO through *in vitro* and *in vivo* experiments. Tetramethylpyrazine is an alkaloid and one of the active components of Chuanxiong, which exerts a wide range of cardiovascular protective effects (Lin et al., 2022). For example, tetramethylpyrazine inhibits isoproterenol-induced cardiomyocyte hypertrophy in neonatal mice by decreasing the expression of CaN (Ji et al., 2014). In a study of ApoE^−/−^ mice fed a high-fat diet, Zhang et al. showed that tetramethylpyrazine (45.05 mg/kg*i. g.*) to improve dyslipidemia by down-regulating progesterone and ADIPOQ receptor 3 and inhibiting the SCAP/SREBP-1c signaling pathway (Zhang et al., 2017). Linarin is one of the active components of Yejuhua. Studies have shown that linarin can regulate gut microbiota, and reduce the production of LPS, thereby inhibiting the expression of TLR4/Myd88 pathway in blood vessels, improving vascular endothelial function, and ultimately reducing BP in hypertensive rats (Wang et al., 2021). In brief, QDT concentrates on the advantages of these herbs and allows these herbs to play a synergistic role in the treatment of hypertension.

In this study, some limitations need to be acknowledged. First, the duration of treatment was only 12 weeks, which limits our ability to assess the medium to long-term effects and safety of QDT in treating essential hypertension. Second, the secondary outcome of 24-h ambulatory BP measures was not analyzed due to poor patient compliance and excessive missing data. Third, this study was conducted exclusively in the Chinese population, and it remains unclear whether the effects of QDT are consistent in other ethnic groups. Lastly, the COVID-19 pandemic resulted in the dropout of many cases. It is hoped that future QDT studies will refine these limitations.

## 5 Conclusion

The efficacy of the combination of QDT and amlodipine besylate is superior to monotherapy with amlodipine besylate in the treatment of essential hypertension. The use of QDT under the premise of syndrome differentiation and treatment holds promise as a potential adjunctive treatment for essential hypertension. However, the above conclusions still require further investigation through rigorous clinical research.

## Data Availability

The original contributions presented in the study are included in the article/Supplementary Material, further inquiries can be directed to the corresponding author.

## References

[B1] Albasri A. Hattle M. Koshiaris C. Dunnigan A. Paxton B. Fox S. E. (2021). Association between antihypertensive treatment and adverse events: Systematic review and meta-analysis. BMJ 372, n189. 10.1136/bmj.n189 33568342 PMC7873715

[B2] Bai Y. Li K. Shao J. Luo Q. Jin L. H. (2018). Flos Chrysanthemi Indici extract improves a high-sucrose diet-induced metabolic disorder in Drosophila. Exp. Ther. Med. 16, 2564–2572. 10.3892/etm.2018.6470 30186490 PMC6122459

[B3] Burnier M. Egan B. M. (2019). Adherence in hypertension. Circ. Res. 124, 1124–1140. 10.1161/CIRCRESAHA.118.313220 30920917

[B4] Chen J. Huang Y. Hu X. Bian X. Nian S. (2021). Gastrodin prevents homocysteine‐induced human umbilical vein endothelial cells injury via PI3K/Akt/eNOS and Nrf2/ARE pathway. J. Cell. Mol. Med. 25, 345–357. 10.1111/jcmm.16073 33320446 PMC7810955

[B5] Chen Z. Zhang C. Gao F. Fu Q. Fu C. He Y. (2018). A systematic review on the rhizome of Ligusticum chuanxiong Hort. (Chuanxiong). Food Chem. Toxicol. Int. J. Publ. Br. Ind. Biol. Res. Assoc. 119, 309–325. 10.1016/j.fct.2018.02.050 29486278

[B6] Chinese Pharmacopoeia Commission (2015). Chinese Pharmacopoeia. Beijing: China Medical Science and Technology Press.

[B7] Hripcsak G. Suchard M. A. Shea S. Chen R. You S. C. Pratt N. (2020). Comparison of cardiovascular and safety outcomes of chlorthalidone vs hydrochlorothiazide to treat hypertension. JAMA Intern. Med. 180, 542–551. 10.1001/jamainternmed.2019.7454 32065600 PMC7042845

[B8] Hu H. Xiao H. Bao H. Li M. Xue C. Li Y. (2020). Tissue distribution comparison of six active ingredients from an eucommiae cortex extract between normal and spontaneously hypertensive rats. Evid.-Based Complement. Altern. Med. ECAM 2020, 2049059. 10.1155/2020/2049059 PMC7298282 32595724

[B9] Huang X. Ngaenklangdon S. He J. Gao X. (2021). Traditional Chinese medicine’s liver yang ascendant hyperactivity pattern of essential hypertension and its treatment approaches: A narrative review. Complement. Ther. Clin. Pract. 43, 101354. 10.1016/j.ctcp.2021.101354 33706064

[B10] Ji X. X. Song X. L. Qian W. Yu X. L. Zhu J. Y. (2014). Effects and mechanism of action of ligustrazine on isoprenaline-induced cardiomyocyte hypertrophy. Cell biochem. Biophys. 70, 1513–1518. 10.1007/s12013-014-0086-2 25027096

[B11] Ji Z. Lin S. Hu H. Sheng X. Yang F. Wang X. (2022). Network meta-analysis of efficacy and safety of oral Chinese patent medicines combined with conventional Western medicine in treatment of hypertension. China J. Chin. Mat. Medica 47, 1955–1988. 10.19540/j.cnki.cjcmm.20211223.501 35534266

[B12] Jiang F. Tiffany C. Guijing W. Fleetwood L. (2020). Association between cost-related medication nonadherence and hypertension management among US adults. Am. J. Hypertens. 33, 879–886. 10.1093/ajh/hpaa072 32369108

[B13] Lacey B. Lewington S. Clarke R. Kong X. L. Chen Y. Guo Y. (2018). Age-specific association between blood pressure and vascular and non-vascular chronic diseases in 0·5 million adults in China: A prospective cohort study. Lancet Glob. Health 6, e641–e649. 10.1016/S2214-109X(18)30217-1 29773120 PMC5960069

[B14] Lai X. Dong Z. Wu S. Zhou X. Zhang G. Xiong S. (2022). Efficacy and safety of Chinese herbal medicine compared with losartan for mild essential hypertension: A randomized, multicenter, double-blind, noninferiority trial. Circ. Cardiovasc. Qual. Outcomes 15, e007923. 10.1161/CIRCOUTCOMES.121.007923 35105177

[B15] Li J. Shu F. Zhang Y. (2019a). To observe the effect of valsartan combined with Qiangli Dingxuan tablets in the treatment of elderly patients with essential hypertension. Cardiovasc. Dis. J. Integr. Tradit. Chin. West. Med. 7, 12–14. 10.16282/j.cnki.cn11-9336/r.2019.07.005

[B16] Li J. Shu F. Zhang Y. (2019b). To observe the effect of valsartan combined with Qiangli Dingxuan tablets in the treatment of elderly patients with essential hypertension. Cardiovasc. Dis. J. Integr. Tradit. Chin. West. Med. 7, 12–14. 10.16282/j.cnki.cn11-9336/r.2019.07.005

[B17] Li Q. Lan T. He S. Chen W. Li X. Zhang W. (2021). A network pharmacology-based approach to explore the active ingredients and molecular mechanism of Lei-gong-gen formula granule on a spontaneously hypertensive rat model. Chin. Med. 16, 99. 10.1186/s13020-021-00507-1 34627325 PMC8501634

[B18] Li Z. Deng X. Huang W. Li L. Li H. Jing X. (2014). Lignans from the bark of Eucommia ulmoides inhibited Ang II-stimulated extracellular matrix biosynthesis in mesangial cells. Chin. Med. 9, 8. 10.1186/1749-8546-9-8 24524265 PMC3937011

[B19] Lin J. Wang Q. Zhou S. Xu S. Yao K. (2022). Tetramethylpyrazine: A review on its mechanisms and functions. Biomed. Pharmacother. 150, 113005. 10.1016/j.biopha.2022.113005 35483189

[B20] Lin J. Zhang J. Duan J. Yao K. (2021). Study on the mechanism of qianglidingxuan tablets in the treatment of hypertension based on network pharmacology and molecular docking. World Chin. Med. 16, 2570–2575. 10.3969/j.issn.1673-7202.2021.17.009

[B21] Liu W. Wang L. Yu J. Asare P. F. Zhao Y.-Q. (2015). Gastrodin reduces blood pressure by intervening with RAAS and PPAR*γ* in SHRs. Evid. Based Complement. Altern. Med. 2015, e828427. 10.1155/2015/828427 PMC4637485 26587048

[B22] Lu J. Lu Y. Wang X. Li X. Linderman G. C. Wu C. (2017). Prevalence, awareness, treatment, and control of hypertension in China: Data from 1·7 million adults in a population-based screening study (China PEACE million persons Project). Lancet lond. Engl. 390, 2549–2558. 10.1016/S0140-6736(17)32478-9 29102084

[B23] Luo H. Fen Z. Li J. Xie Y. (2022). The establishment of HPLC characteristic chromatogram analysis method for Qiangli Dingxuan tablets. J. Northwest Univ. Nat. Sci. Ed. 52, 90–98. 10.16152/j.cnki.xdxbzr.2022-01-011

[B24] Luo L. Wu W. Zhou Y. Yan J. Yang G. Ouyang D. (2010). Antihypertensive effect of Eucommia ulmoides Oliv. extracts in spontaneously hypertensive rats. J. Ethnopharmacol. 129, 238–243. 10.1016/j.jep.2010.03.019 20347950

[B25] Matias M. Silvestre S. Falcão A. Alves G. (2016). Gastrodia elata and epilepsy: Rationale and therapeutic potential. Phytomedicine Int. J. Phytother. Phytopharm. 23, 1511–1526. 10.1016/j.phymed.2016.09.001 27765372

[B26] Shira B. Keren M.-G. Ilan A. Ben W. Zev Moshe S. (2015). Angiotensin-converting enzyme inhibitor-induced angioedema. Am. J. Med. 128, 120–125. 10.1016/j.amjmed.2014.07.011 25058867

[B27] Sun P. Wang M. Li Z. Wei J. Liu F. Zheng W. (2022). Eucommiae cortex polysaccharides mitigate obesogenic diet-induced cognitive and social dysfunction via modulation of gut microbiota and tryptophan metabolism. Theranostics 12, 3637–3655. 10.7150/thno.72756 35664075 PMC9131264

[B28] Wang J. Xiong X. Liu W. (2014). Traditional Chinese medicine syndromes for essential hypertension: A literature analysis of 13,272 patients. Evid.-Based Complement. Altern. Med. ECAM 2014, 418206. 10.1155/2014/418206 PMC3934631 24660016

[B29] Wang Y.-J. Su J. Yu J.-J. Yan M.-Q. Shi M.-L. Huang Q.-D. (2021). Buddleoside-rich Chrysanthemum indicum L. Extract has a beneficial effect on metabolic hypertensive rats by inhibiting the enteric-origin LPS/TLR4 pathway. Front. Pharmacol. 12, 755140. 10.3389/fphar.2021.755140 34690786 PMC8532163

[B30] Wang Y. (2020). Effect comparison between amlodipine besylate and levamlodipine besylate in the treatment of hypertension. China Mod. Med. 27, 130–133.

[B31] Wang Z. Chen Z. Zhang L. Wang X. Hao G. Zhang Z. (2018). Status of hypertension in China: Results from the China hypertension Survey, 2012-2015. Circulation 137, 2344–2356. 10.1161/CIRCULATIONAHA.117.032380 29449338

[B32] Writing Group of 2018 Chinese Guidelines for the Management of Hypertension (2019). 2018 Chinese guidelines for the management of hypertension. Chin. J. Cardiovasc. Med. 24, 24–56.

[B33] Xiong X. Wang P. Li X. Zhang Y. (2015). Shenqi pill, a traditional Chinese herbal formula, for the treatment of hypertension: A systematic review. Complement. Ther. Med. 23, 484–493. 10.1016/j.ctim.2015.04.008 26051584

[B34] Xiong X. Yang X. Liu Y. Zhang Y. Wang P. Wang J. (2013). Chinese herbal formulas for treating hypertension in traditional Chinese medicine: Perspective of modern science. Hypertens. Res. Off. J. Jpn. Soc. Hypertens. 36, 570–579. 10.1038/hr.2013.18 PMC3703711 23552514

[B35] Xu C. (2022). To observe the clinical effect of Qiangli Dingxuan tablet combined with flunarizine hydrochloride capsule in the treatment of vertigo. J. Math. Med. 35, 287–289. 10.3969/j.issn.1004-4337.2022.02.043

[B36] Xu J. Mao L. Zhao W. Fu S. Ding Y. (2021). Effects of buyang huanwu decoction and Qiangli dingxuan tablet on vertigo symptoms, oxidative stress and blood flow index in poaterior circulation ischemia vertigo patients. Prog. Mod. Biomed. 21, 463–466+417. 10.13241/j.cnki.pmb.2021.03.012

[B37] Yang T.-Y. Wu Y.-L. Yu M.-H. Hung T.-W. Chan K.-C. Wang C.-J. (2022). Mulberry leaf and neochlorogenic acid alleviates glucolipotoxicity-induced oxidative stress and inhibits proliferation/migration via downregulating ras and FAK signaling pathway in vascular smooth muscle cell. Nutrients 14, 3006. 10.3390/nu14153006 35893859 PMC9331252

[B38] Yin P. Yan P. (2021). Clinical study of Qiangli Dingxuan tablets combined with candesartan in the treatment of essential hypertension. Chin. Remedies Clin. 21, 271–273. 10.11655/zgywylc2021.02.033

[B39] Zhang D.-Y. Cheng Y.-B. Guo Q.-H. Shan X.-L. Wei F.-F. Lu F. (2020). Treatment of masked hypertension with a Chinese herbal formula: A randomized, placebo-controlled trial. Circulation 142, 1821–1830. 10.1161/CIRCULATIONAHA.120.046685 33019798

[B40] Zhang S. Xiong X. Duan J. Wang Q. Li C. Yao K. (2022). Oncolytic adenoviruses synergistically enhance anti-PD-L1 and anti-CTLA-4 immunotherapy by modulating the tumour microenvironment in a 4T1 orthotopic mouse model. Beijing J. Tradit. Chin. Med. 41, 456–465. 10.1038/s41417-021-00389-3 PMC9113929 34561555

[B41] Zhang Y. Ouyang K. Chen N. Wu N. (2021). Clinical observation of Qiangli dingxuan tablet combined with citalopram in treatment of persistent posture - perception dizziness. Chin. Arch. Tradit. Chin. Med. 39, 225–227. 10.13193/j.issn.1673-7717.2021.02.057

[B42] Zhang Y. Ren P. Kang Q. Liu W. Li S. Li P. (2017). Effect of tetramethylpyrazine on atherosclerosis and SCAP/SREBP-1c signaling pathway in ApoE-/- mice fed with a high-fat diet. Evid.-Based Complement. Altern. Med. ECAM 2017, 3121989. 10.1155/2017/3121989 PMC5405370 28491104

[B43] Zhao D. Liu J. Wang M. Zhang X. Zhou M. (2019). Epidemiology of cardiovascular disease in China: Current features and implications. Nat. Rev. Cardiol. 16, 203–212. 10.1038/s41569-018-0119-4 30467329

[B44] Zhao S. (2018). Effect of Qiangli Dingxuan tablets plus nifedipine on blood pressure and vertigo symptoms in patients with hypertension. Chin. J. Integr. Med. Cardio-Cerebrovascuiar Dis. 16, 2746–2748. 10.12102/j.issn.1672-1349.2018.18.048

[B45] Zheng X. (2002). Instructional principle of the latest Chinese herbal medicine to Clinical Research. Beijing: The Medicine Science and Technology Press of China.

