# Supplementary material for: Efficacy and safety of Qiangli Dingxuan tablet combined with amlodipine besylate for essential hypertension: a randomized, double-blind, placebo-controlled, parallel-group, multicenter trial
Source: Front Pharmacol. 2023 Jul 10;14:1225529. doi: 10.3389/fphar.2023.1225529 (PMC10363978; doi:10.3389/fphar.2023.1225529)
Supplement: Supplementary file 1 [file DataSheet1.docx]

Supplementary Material

Efficacy and Safety of Qiangli Dingxuan Tablet Combined with Amlodipine Besylate for Essential Hypertension: A Randomized, Double‑Blind, Placebo‑Controlled, Parallel‑Group, Multicenter Trial

**Jianguo Lin ^1, 2, †^, Qingqing Wang ^1, †^, Dongsheng Zhong ^1^, Jinju Zhang ^1^, Tianhui Yuan ^3^, Hui Wu ^3^, Bin Li^4^, Shuangdi Li^5^, Xiaoliu Xie ^6^, Dongqing An^6^, Yue Deng ^5^, Shaoxiang Xian ^3^, Xingjiang Xiong ^1^, Kuiwu Yao ^1, 7, *^**

^1^ Guang’anmen Hospital, China Academy of Chinese Medical Sciences, Beijing, China

^2^Tianjin University of Traditional Chinese Medicine, Tianjin, China

^3^First Affiliated Hospital of Guangzhou University of Chinese Medicine, Guangzhou, China

^4^First Affiliated Hospital of Henan University of Chinese Medicine, Zhengzhou, China

^5^Affiliated Hospital of Changchun University of Chinese Medicine, Changchun, China

^6^ Traditional Chinese Medicine Hospital of Xinjiang Uygur Autonomous Region, Urumqi, China

^7^ Eye Hospital China Academy of Chinese Medical Sciences, Beijing, China

**^†^These authors have contributed equally to this work and share first authorship**

**^*^ Correspondence:** Kuiwu Yao; [yaokuiwu@126.com](mailto:yaokuiwu@126.com)

# HPLC fingerprints of Qiangli Dingxuan tablet

## Reagents and Drugs

10 batches of Qiangli Dingxuan tablet (QDT)samples, batch numbers were DX078907 (S1), DX079907 (S2), DX080907 (S3), DX081907 (S4), DX082908 (S5), DX083908 (S6), DX084908 (S7), DX085908 (S8), DX086908 (S9) and DX087908 (S10) were provided by Shaanxi Hanwang Pharmaceutical Co., Ltd, Shaanxi, China.

Reference substance: Gastrodin (HR16313B1), 5-hydroxymethyl furfural (HH248894198), p-hydroxybenzyl alcohol (H21D6Q7813), neochlorogenic acid (HR20422B1), p-hydroxybenzaldehyde (HA060208198), pinoresinol diglucoside (HS0870W2), ligustilide (HL09355198) were purchased from Baoji Chenguang Biotechnology Co., Ltd. Chlorogenic acid (110753-201415), linarin (111528-201300), vanillic acid (110776-200402) and sophoroside (111695-200501) were purchased from China Institute for Food and Drug Control. Gallic acid (CHB180114) was purchased from Chengdu Cloma Biotechnology Co., Ltd.

## The chromatographic conditions

Chromatographic conditions: Lamdo Stamsil C_18_ column (250 × 4.6 mm, 5 μm); Mobile phase acetonitrile (A) -0.05% aqueous phosphoric acid (B), gradient elution (0-5 min, 5%-7% A; 5 ~ 15 min, 7% A; 15-30 min, 7%-12% A; 30-90 min, 12%-20% A; 90-100 min, 20%-50% A); Detection wavelength: 280 nm; Injection volume: 5μL; Volume flow: 0.8 mL/min; Column temperature: 30℃.

## Results

10 batches of QDT chromatograms were imported into the "Traditional Chinese Medicine Fingerprints Similarity System (2004 A version)" to obtain the HPLC fingerprint of QDT and the control R(Figs. 1, 2). 10 batches of QDT samples were matched with 34 common peaks, and the similarity was above 0.9, indicating that the quality was relatively stable and the common components were basically the same (Table 1).


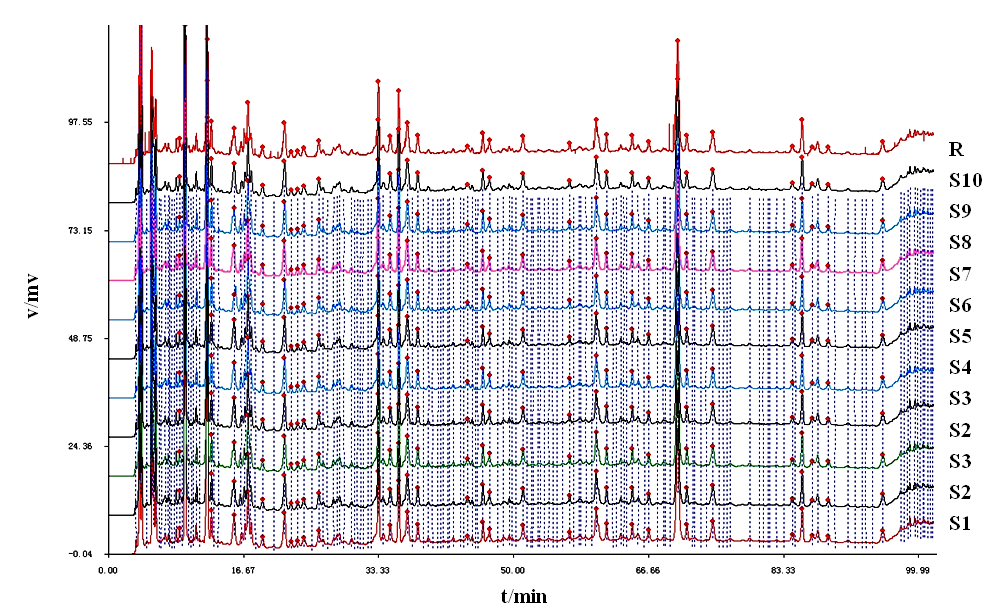


**Fig. 1. HPLC fingerprints of 10 batches of samples.** R: fitted fingerprint; S1-S10: 10 batches of QDT fingerprint.

**
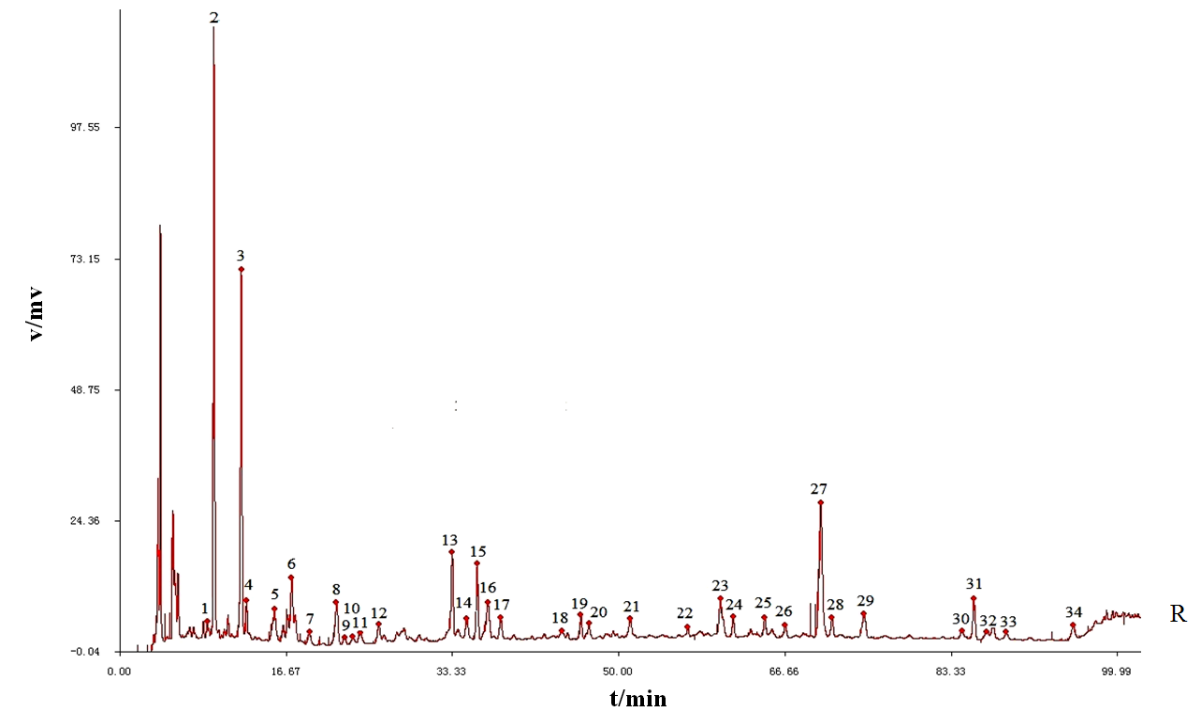
**

**Fig. 2. HPLC fingerprints of the control R.** 1～34：Characteristic fingerprints peak；1. Gastrodin；2. Gallic acid；3. 5-hydroxymethyl furfural；5. p-Hydroxybenzyl alcohol；8. Neochlorogenic acid；13. Chlorogenic acid；14. Vanillic acid；16. p-Hydroxybenzaldehyde；20. Pinoresinol Diglucoside；26. Sophoricoside；27. Ligustilide；31. Linarin.

**Table.1 Similarity evaluation of 10 batches of samples**

|  |  | S1 | S2 | S3 | S4 | S5 | S6 | S7 | S8 | S9 | S10 | R |
| --- | --- | --- | --- | --- | --- | --- | --- | --- | --- | --- | --- | --- |
| S1 |  | 1.000 | 0.992 | 0.994 | 0.926 | 0.968 | 0.950 | 0.965 | 0.964 | 0.947 | 0.973 | 0.991 |
| S2 |  | 0.992 | 1.000 | 0.993 | 0.919 | 0.967 | 0.935 | 0.953 | 0.960 | 0.946 | 0.969 | 0.989 |
| S3 |  | 0.994 | 0.993 | 1.000 | 0.930 | 0.966 | 0.948 | 0.962 | 0.960 | 0.938 | 0.973 | 0.991 |
| S4 |  | 0.926 | 0.919 | 0.930 | 1.000 | 0.945 | 0.906 | 0.940 | 0.925 | 0.964 | 0.900 | 0.934 |
| S5 |  | 0.968 | 0.967 | 0.966 | 0.945 | 1.000 | 0.934 | 0.961 | 0.969 | 0.925 | 0.942 | 0.976 |
| S6 |  | 0.950 | 0.935 | 0.948 | 0.906 | 0.934 | 1.000 | 0.961 | 0.935 | 0.931 | 0.946 | 0.958 |
| S7 |  | 0.965 | 0.953 | 0.962 | 0.94 | 0.961 | 0.961 | 1.000 | 0.969 | 0.930 | 0.960 | 0.976 |
| S8 |  | 0.964 | 0.960 | 0.960 | 0.925 | 0.969 | 0.935 | 0.969 | 1.000 | 0.939 | 0.972 | 0.980 |
| S9 |  | 0.947 | 0.946 | 0.938 | 0.964 | 0.925 | 0.931 | 0.930 | 0.939 | 1.000 | 0.959 | 0.956 |
| S10 |  | 0.973 | 0.969 | 0.973 | 0.900 | 0.942 | 0.946 | 0.960 | 0.972 | 0.959 | 1.000 | 0.983 |
| R |  | 0.991 | 0.989 | 0.991 | 0.934 | 0.976 | 0.958 | 0.976 | 0.980 | 0.956 | 0.983 | 1.000 |


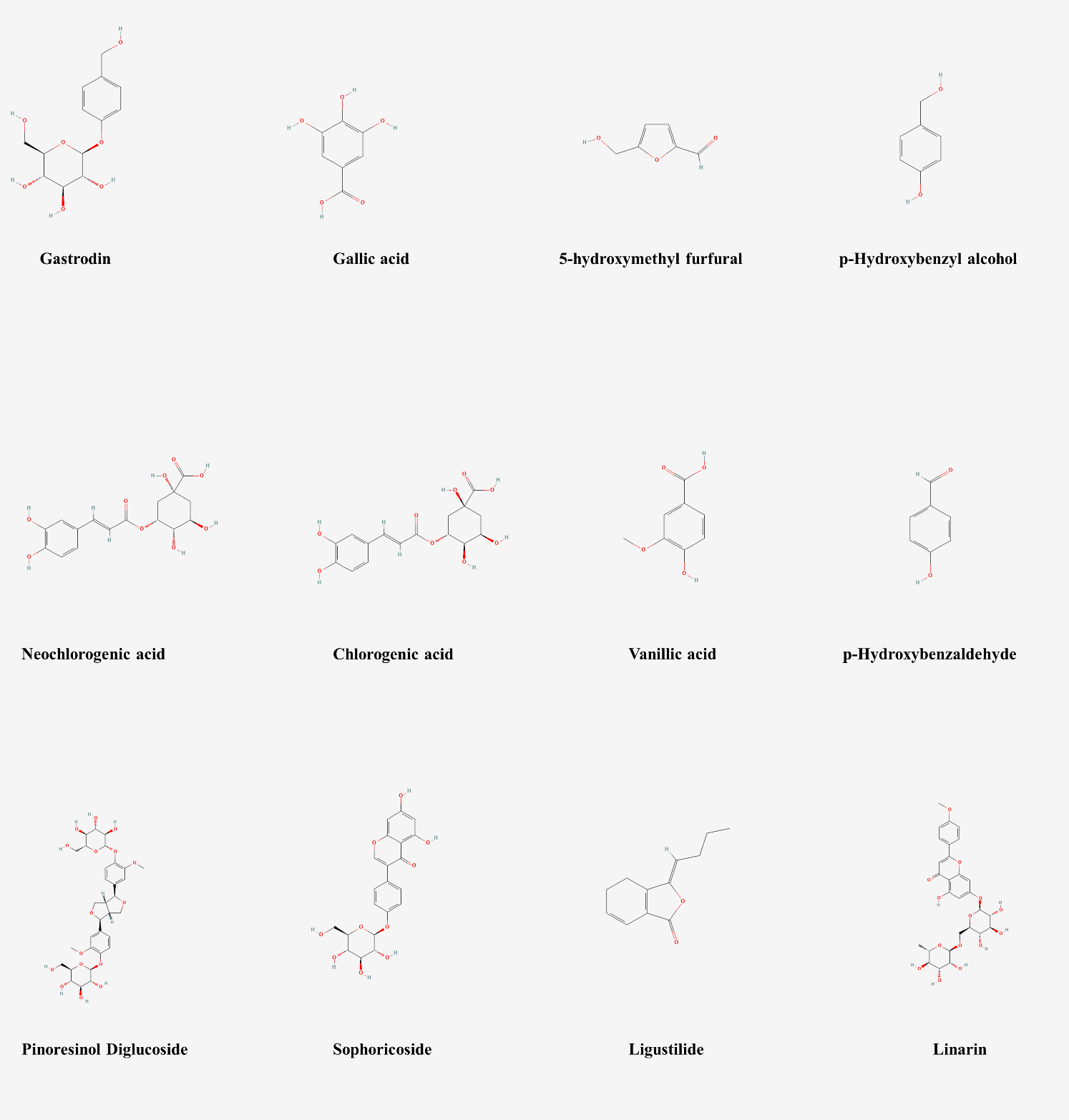


**Fig. 3.** The chemical structure of active ingredients of Qiangli Dingxuan tablet.

## References

Luo, H., Fen, Z., Li, J., Xie, Y., et al., 2022. The establishment of HPLC characteristic chromatogram analysis method for Qiangli Dingxuan tablets. J. Northwest Univ. Nat. Sci. Ed. 52, 90–98.

# Traditional Chinese Medicine Syndrome Score Scale

| **Traditional Chinese Medicine Syndrome Score Scale** | | | | |
| --- | --- | --- | --- | --- |
| **Syndrome** | **1（mild）** | **2（moderate）** | **3（severe）** |  |
| Dizziness |  |  |  |  |
| Headache |  |  |  |  |
| Impetuosity |  |  |  |  |
| Aching lumbus |  |  |  |  |
| Limp knees |  |  |  |  |
| Sphoria with feverish sensation in chest,palms and soles |  |  |  |  |
| Head heavy as if swathed |  |  |  |  |
| Chest stuffiness |  |  |  |  |
| Vomiting of phlegm-drool |  |  |  |  |
| Chilly sensation and the cold limbs |  |  |  |  |
| Flushed face |  |  |  |  |
| Red eyes |  |  |  |  |
| Dry mouth |  |  |  |  |
| Bitterness in the mouth |  |  |  |  |
| Constipation |  |  |  |  |
| Hematuria |  |  |  |  |
| Palptation |  |  |  |  |
| Insomnia |  |  |  |  |
| Tinnitus |  |  |  |  |
| Amnesia |  |  |  |  |
| Bland taste in the mouth |  |  |  |  |
| Low food intake |  |  |  |  |
| Shortness of breath |  |  |  |  |
| Nycturia |  |  |  |  |

# Safety evaluation

**Table 1.** Laboratory parameters (analysis based on safety set).

| **Outcomes** | **Visit** | **QDT group** | | **Placebo group** | | ***P* value** |
| --- | --- | --- | --- | --- | --- | --- |
|  |  | **n (missing)** | **Mean (SD)/ n (%)** | **n (missing)** | **Mean (SD)/ n (%)** |  |
| **Blood routine test** | | | | | | |
| HGB, g/L | Baseline | 124(9) | 147.66 (15.19) | 127(8) | 148.78 (13.65) | 0.4893 |
|  | 12 weeks | 90(43) | 146.50 (15.55) | 94(41) | 144.45 (15.35) | 0.4938 |
| RBC, 10^12^/L | Baseline | 124(9) | 4.85 (0.43) | 127(8) | 4.84 (0.46) | 0.6325 |
|  | 12 weeks | 90(43) | 4.73 (0.60) | 94(41) | 4.66 (0.57) | 0.3387 |
| WBC, 10^9^/L | Baseline | 124(9) | 6.60 (1.68) | 127(8) | 6.72 (2.03) | 0.8503 |
|  | 12 weeks | 90(43) | 6.70 (1.52) | 94(41) | 6.86 (1.99) | 0.9569 |
| NEUT, 10^9^/L | Baseline | 124(9) | 3.90 (1.28) | 127(8) | 3.71 (1.15) | 0.1334 |
|  | 12 weeks | 90(43) | 4.01 (1.62) | 94(41) | 4.13 (1.85) | 0.9901 |
| LY, % | Baseline | 124(9) | 33.41 (8.03) | 127(8) | 35.37 (8.06) | 0.0547 |
|  | 12 weeks | 90(43) | 33.97 (7.56) | 94(41) | 35.39 (9.41) | 0.3586 |
| PLT, 10^9^/L | Baseline | 124(9) | 240.79 (75.55) | 127(8) | 245.89 (60.92) | 0.4630 |
|  | 12 weeks | 90(43) | 240.42 (71.47 | 94(41) | 243.99 (61.90) | 0.8713 |
| **Liver function test** | | | | | | |
| ALT, U/L | Baseline | 126(7) | 23.57 (9.24) | 132(3) | 24.12 (11.88) | 0.6426 |
|  | 12 weeks | 90(43) | 20.94 (9.32) | 94(41) | 20.31 (8.98) | 0.4914 |
| AST, U/L | Baseline | 128(5) | 22.11 (5.70) | 134(1) | 22.16 (6.76) | 0.5692 |
|  | 12 weeks | 90(43) | 20.58 (5.02) | 94(41) | 21.31 (5.62) | 0.5360 |
| TBIL, μmol/L | Baseline | 126(7) | 14.78 (4.49) | 129(6) | 14.97 (5.48) | 0.6200 |
|  | 12 weeks | 90(43) | 14.12 (4.66) | 94(41) | 14.53 (4.79) | 0.3055 |
| DBIL, μmol/L | Baseline | 125(8) | 2.53 (1.01) | 128(7) | 2.62 (1.18) | 0.7193 |
|  | 12 weeks | 90(43) | 2.56 (0.89) | 94(41) | 2.81 (1.36) | 0.2231 |
| TP, g/L | Baseline | 127(6) | 72.86 (3.93) | 129(6) | 73.17 (4.86) | 0.6509 |
|  | 12 weeks | 90(43) | 72.01 (7.32) | 94(41) | 72.38 (6.16) | 0.7765 |
| ALP, U/L | Baseline | 117(16) | 78.36 (23.31) | 117(18) | 80.97 (21.14) | 0.1887 |
|  | 12 weeks | 90(43) | 79.17 (21.94) | 93(42) | 80.28 (22.20) | 0.6612 |
| **Kidney function test** | | | | | | |
| BUN, mmol/L | Baseline | 131(2) | 5.24 (1.17) | 127(8) | 5.25 (2.11) | 0.2962 |
|  | 12 weeks | 90(43) | 5.11 (1.42) | 94(41) | 5.03 (1.36) | 0.6388 |
| Scr, μmol/L | Baseline | 131(2) | 66.05 (14.63) | 129(6) | 65.92 (13.68) | 0.9205 |
|  | 12 weeks | 90(43) | 67.47 (28.09) | 94(41) | 64.91 (14.45) | 0.7722 |
| BUA, μmol/L | Baseline | 131(2) | 347.55 (88.05) | 129(6) | 350.44 (82.23) | 0.6752 |
|  | 12 weeks | 90(43) | 341.47 (89.29) | 94(41) | 343.87 (90.10) | 0.9713 |
| **Urine routine test** | | | | | | |
| Urine protein, ≥（+） | Baseline | 127(6) | 6(4.72) | 127(8) | 7(5.51) | 0.6324 |
|  | 12 weeks | 123(10) | 2(1.62) | 111(24) | 4(3.60) | 0.3231 |
| Urine glucose, ≥（+） | Baseline | 127(6) | 2(1.57) | 127(8) | 1(0.79) | 0.6399 |
|  | 12 weeks | 123(10) | 2(1.62) | 111(24) | 3(2.70) | 0.2972 |
| Urine erythrocyte, ≥（+） | Baseline | 127(6) | 18(14.17) | 127(8) | 19(14.96) | 0.5954 |
|  | 12 weeks | 123(10) | 8(6.50) | 111(24) | 10(9.01) | 0.4084 |
| Urine leukocyte, ≥（+） | Baseline | 127(6) | 15(11.81) | 127(8) | 15(11.81) | 0.5907 |
|  | 12 weeks | 123(10) | 8(6.50) | 111(24) | 11(9.91) | 0.3521 |

HGB, hemoglobin; RBC, red blood cell count; WBC, white blood cell count; PLT, blood platelet count; NEUT, neutrophil count; LY, lymphocyte; ALT, glutamic pyruvic transaminase; AST, glutamic oxalacetic transaminase; TBIL, total bilirubin; DBIL, direct bilirubin; TP, total protein; ALP, alkaline phosphatase; BUN, blood urea nitrogen; Scr, serum creatinine; BUA, blood uric acid.
